# Understanding the Heterogeneity of Resident Liver Macrophages

**DOI:** 10.3389/fimmu.2019.02694

**Published:** 2019-11-19

**Authors:** Camille Blériot, Florent Ginhoux

**Affiliations:** ^1^Singapore Immunology Network (SIgN), Agency for Science, Technology and Research (A*STAR), Singapore, Singapore; ^2^Shanghai Institute of Immunology, School of Medicine, Shanghai Jiao Tong University, Shanghai, China; ^3^Translational Immunology Institute, SingHealth Duke-NUS Academic Medical Centre, Singapore, Singapore

**Keywords:** liver, macrophage, heterogeneity, single cell RNA sequencing, monocyte

## Abstract

Resident tissue macrophages (RTMs) are cells with a high functional plasticity assuming pleiotropic roles in their tissue of residence, from clearance of dead cells and metabolic sensing in steady state to cytokine production and tissue repair during inflammation. The liver has long been considered as only populated by Kupffer cells (KCs), a macrophage population assumed to be in charge of all of these functions. However, we know now that KCs are not the only macrophage population in the liver, that recently was shown to contain also capsular macrophages, monocyte-derived macrophages as well as recruited peritoneal macrophages inherited from previous inflammatory events. These macrophages exhibit different origins, time of establishing residence and locations in the liver, with both ontogenical and environmental factors shaping their identity and functions. Furthermore, liver macrophages reside in a complex environment with a pronounced metabolic zonation. Here, we briefly discuss how these intrinsic and extrinsic factors influence macrophage biology and liver physiology in general. We notably focus on how the recent advances of single cell transcriptomic approaches are changing our understanding of liver macrophages and diseases.

## Introduction

The liver is known to assume a large repertoire of diverse functions such as detoxication of numerous metabolites, synthesis of essential proteins, or recycling of iron-containing red blood cells (1). Such versatility renders this organ indispensable for a healthy physiological state knowing that the only treatment of liver failure remains limited to organ transplantation. Liver and notably the hepatocytes, its fundamental metabolic units, can be affected by numerous pathologies among which are hepatitis, steatosis, cirrhosis, or hepatocarcinoma (1). Etiology of these distinct diseases is complex and involves genetic and environmental factors yet difficult to stratify in a comprehensive manner. Furthermore, at a mechanistic level, development of a liver pathology such as fibrosis for example not only implies hepatocytes but also the other liver cell populations in the forefront of which are macrophages, but also stellate and endothelial cells (2). So, understanding the relationships that are established between these different essential cellular liver components appear necessary to better understand liver functions and pathologies.

## Diversity of Liver Macrophages: Origin and Location Matter

**Figure 1:**
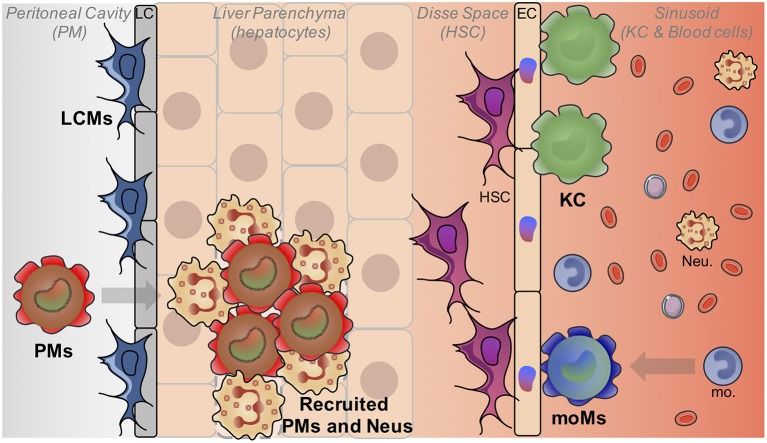
Liver macrophage heterogeneity. The liver is populated by different macrophage populations. The most abundant one is composed by embryonically-derived Kupffer cells (KC) which reside in liver sinusoids and interact mainly with HSC and EC. Monocyte-derived macrophages (MoM) can also acquire a KC-like phenotype after inflammation. Liver capsular macrophages (LCM) are present at the level of the liver capsule (LC). Finally, mature peritoneal macrophages (PMs) can also be recruited in the liver notably in case of injuries in the parenchyma. (Neu, Neutrophils).

Macrophages represent by far the most abundant immune cells in the liver. Hepatic macrophages are still often referred as Kupffer cells (KCs) that indeed represent the major fraction of liver macrophages. KCs were first described more than one century ago by Kupffer who initially described them as endothelial cells, components of liver vascular walls (3). Then, a few years later, they were correctly reassigned as macrophages by Browicz (4). Indeed, even if KCs are located in the liver, they do not reside in the parenchyma and are not in direct contact with the hepatocytes, as they are located within the liver sinusoids where they are in contact with the blood compartment (Figure 1). KCs were then included in the mononuclear phagocyte system by Van Furth et al. (5) and considered thereafter as the liver-resident monocyte-derived macrophages. But numerous recent studies using notably powerful fate-mapping models have completely revisited the dogma of the monocytic origin of many resident macrophages, including KCs (6–11). It is now clearly established that KCs do not derive from adult circulating monocytes but rather from fetal liver monocytic precursors that expand and maintain themselves during the entire life of the organism (8–10). This renders KC renewal almost independent of bone-marrow derived cells at steady state.

Besides of the embryonically-derived KC population that represents the vast majority of liver macrophages, at least at steady-state, another population of macrophages residing in the hepatic capsule has been recently described (12). These liver capsular macrophages (LCMs) are phenotypically and developmentally different from KCs. Indeed, although expressing typical macrophage markers such as CD64 and F4/80, LCMs are negative for the canonical KC markers Tim4 and Clec4F, and express rather markers traditionally expressed by dendritic cells such as MHCII and CD11c (12, 13). Moreover, LCMs do not derive from embryonic precursors but arise from adult circulating monocytes. Whether such LCMs represent an homogeneous population or comprised subsets of macrophages and dendritic cells remain to be established.

In addition to these two main macrophage populations, the liver may contain a variable amount of recruited blood monocyte-derived macrophages. Indeed, in several inflammatory conditions and notably when KC depletion occurs, replacement by monocyte-derived macrophages can be observed (14, 15). Some of the newly-recruited cells will acquire a similar transcriptomic pattern with time and resolution of inflammation and will establish residence in the liver, assuming similar functions than the original macrophages (15). Interestingly, even if adult monocyte-derived cells represent a very minor fraction of liver macrophages in mice grown in pathogen-free facilities with controlled diets, the situation could be very different in humans which are exposed to a more challenging and diverse environment with notably a plethora of foodborne entero-pathogens and various diets. Each infection, even minor and without triggering any detectable symptoms, could induce monocyte recruitment in the liver with few of them differentiating in monocyte-derived macrophages, as observed in the lung (16). This process could be regarded as an immune scar in the liver, each individual having his own immune history shaped by his past infections but also his genetic identity. So in this context, the notion of steady-state appears very restricted to laboratory mice and hardly transposable to healthy humans. In addition, as human fate-mapping models are lacking, although attempt in the single cell genomic era might soon provide answers (17), the origin (embryonic vs. monocyte-derived) of human macrophages is less understood.

Finally, it has also been shown that murine mature peritoneal macrophages could rapidly invade the liver after an injury (18). By using a model of sterile inflammation induced by thermal injury in the liver, the authors have shown that fully differentiated F4/80^hi^ GATA6^+^ peritoneal macrophages migrated to the site of injury. This non-vascular recruitment was mediated by ATP released from dead cells acting as a damage-associated molecular pattern and involved also the Hyaluronan-CD44 interaction. Recruited peritoneal macrophages are responsible for disassembling the necrotic nuclei of dead cells and authors have shown that depletion of peritoneal macrophages significantly delayed the wound healing process (18). Whether these macrophages can maintain themselves in a long-term manner and can become fully integrated in the liver macrophage network as well as the relevance of this phenomenon in human diseases remain to be determined. A table summarizing the phenotypes of these different liver macrophage populations is provided (Table 1) as a comparison with other known liver myeloid populations.

**Table 1:** Phenotype of liver phagocyte populations.

**Markers (mouse)**	**Macrophages**				**Monocytes**	**Dendritic cells**	**Neutrophils**
	**Kupffer cells**	**Monocyte-derived liver macs**	**Capsular macs**	**Peritoneal macs**			
CD11b	+	++ to +	+	**++**	**++**	+	**++**
CD11c	–	–	+	–	–	**++**	–
CD64	+	++ to +	+	+	**++**	+	+
Clec4F	**++**	**– to** **++**	–	–	–	–	–
CX3CR1	–	+ to –	**++**	–	+	–	–
F4/80	**++**	**– to** **++**	**++**	**++**	–	–	–
Ly6C	+	++ to +	+	+	**++**	+	**++**
Ly6G	–	–	–	–	–	–	**++**
MHCII	+	+	**++**	+	+	**++**	–
Tim4	**++**	**– to** **++** **(slowly)**	–	+	–	–	–

*The different populations of liver phagocytes can be resolved by using a panel of common myeloid cell markers. This list of murine markers is not exhaustive but allows enough resolution to identify these populations. Of note, this separation is not accurate for human samples, as mouse-restricted markers such as F4/80 and Clec4F cannot be used, ontogeny remains unclear and the existence of all the subpopulations described herein has not yet been confirmed. Bold values indicate the positive markers that can be used for an efficient gating strategy*.

## Understanding Hepatic Macrophages through their Niche of Residence

Different populations of liver macrophages reside in distinct hepatic niches and are therefore exposed to a different microenvironment. It has now been demonstrated that macrophage homeostasis is tightly controlled by tissue-specific and niche-specific signals (19–22). Macrophages are known to be sessile and self-renewing cells (23) implying that they are solely in direct and intimate interactions with only few tissue cells, allowing profound relationship to be established from the first stages of development (24). An exciting question is to understand in the most exhaustive manner how these cells interact together and with the other components of the liver tissue to shape macrophage identity and functions.

Functionally, the liver is organized in metabolic units called acini. The bloodstream flows from the portal vein and hepatic artery, and circulates through the sinusoids toward the central vein. Hepatocytes represent between 60 and 70% of liver cells. It is known for decades that hepatocytes are heterogeneous with a differential production of enzymes along the portal-central axis resulting in a metabolic zonation (25, 26). Therefore, it has been proposed that oxidative energy metabolism, β-oxidation, amino acid catabolism were mostly performed in the portal zone whereas glycolysis and lipogenesis took place predominantly in the central zone (25). The decreasing oxygen gradient that is established between the blood arriving in the periportal area and leaving by the central vein is obviously one of the key determining factors of the metabolic zonation (27). A genome-wide description of this phenomenon has recently been established by measuring the transcriptomes of thousands of hepatocytes (28) and confirmed at the protein level (29). The same group has also identified zonation at the level of endothelial cells by using paired-cell RNA sequencing, an innovative strategy allowing the profiling of endothelial cells attached to hepatocytes (30). This co-zonation between genes and functions across hepatocytes and endothelial cell was also confirmed recently in humans (31). Whether such zonation impacts KC phenotype, gene expression profile, and functions remains to be investigated.

The other main population of liver-resident cells, even often overlooked by immunologists are the hepatic stellate cells (HSC). These pericytes are localized in the space of Disse between hepatocytes and endothelial cells (Figure 1). They are mainly known for their vitamin A storage function at resting state (32) but they are also known to drive fibrosis by the production of extracellular matrix once activated and trans-differentiated into myofibroblasts (33). Even if HSCs have been less characterized so far, a zonation of these cells has been reported in porcine livers based essentially on morphological criteria (34, 35). Furthermore, a certain level of heterogeneity of HSCs has been observed in an healthy, non-injured mouse liver (36). A most recent contribution has focused on the heterogeneity of HSCs, quiescent at steady-state or activated in a chemically induced model of fibrosis (37). Interestingly, this study mainly reveals heterogeneity for myofibroblasts in fibrotic contexts. Finally, recent studies have elegantly deciphered how these different cells, hepatocytes, endothelial cells and HSCs efficiently collaborate to drive recruited monocyte transition to macrophages in a specific KC depletion model (38, 39). Authors have notably revealed the crucial role of the DLL4-Notch pathway for the programing of recruited monocytes by liver endothelial cells. This activation results in the production of LXRα, a crucial transcription factor involved in the induction and maintenance of KC identity. In parallel of this, HSCs were shown to produce the macrophage colony stimulating factor and hepatocytes to induce expression by monocytes of ID3 (38), a key transcription factor shown to drive fetal liver monocyte to mature KC transition (11). These studies revealed also that such transition from monocyte to monocyte-derived KC was unexpectedly fast, with the different cells producing complementary signals within hours after recruitment allowing monocytes to acquire their new tissue resident identity.

Altogether, even if at a macroscopic level, liver lobules appear homogeneous with a relatively simple tridimensional architecture, they hide a complexity shaped by many factors that should be taken into account to decipher liver macrophage biology and their potential heterogeneity.

## New Approaches to Improve our Understanding of Liver Macrophages

So far, most of the studies dealing with liver macrophages have used very few but specific markers such as CD45 (pan-leukocyte marker), CD11b (pan-myeloid marker) and F4/80 (pan-macrophage marker in mice) to study macrophages, most often assimilated to KCs. While it has been very useful to extend our knowledge on KC biology, this conventional approach consisting in defining populations of interest based on the expression of limited markers by flow cytometry appears more and more outdated nowadays. Discoveries of the distinct ontogeny of KCs and monocyte-derived macrophages and the complexity of the liver niche have challenged the view of liver macrophages as a uniform F4/80+ cell population.

The very recent burst in single cell transcriptomics is indeed profoundly reshaping our approaches to solve key questions in immunology (40). This technology offers the obvious advantage to get access to the expression of thousands of genes at the single cell level instead of a handful of markers which may be highly selective, but which nevertheless remain limited. But the most valuable feature of single cell transcriptomics is the unbiased approach that it is offering. Herein, the most meaningful parameters are not the ones previously anticipated but may be completely unexpected ones, designated in an objective manner by unbiased algorithms. Accordingly, so far, most of the cell populations that have been deeply analyzed turn out to be much more heterogeneous than previously anticipated in every organs, with the existence of overlooked clusters with their own identity and functions (22, 41).

In the liver, the idea of the coexistence of different subsets of KCs has been already proposed (42, 43). Of note, these observations were made by using bone marrow chimeras, an irradiation murine model in which there is a huge recruitment of inflammatory monocytes in the liver giving rise to monocyte-derived macrophages. So as in the studies using the specific KC depletion model aforementioned (38, 39), the irradiated liver undergoes a damage resulting in an inflammatory reaction and this context should therefore be considered different than the steady-state. Interestingly, it has been very recently shown that a subset of the embryonically-derived KCs resists to lethal irradiation, through *cdkn1a* upregulation. This radio-resistance property is lost when native KCs are replaced by their monocyte-derived counterparts, showing clearly that ontogeny contribute to macrophage functional heterogeneity (44). Others have also successfully used the mass cytometry to analyze liver macrophages and notably described two subsets of KCs different from infiltrating monocytes (13). Whether these populations represent ontogenetically independent subsets or distinct activation stages residing in specific locations remains to be established.

Single cell transcriptomic approaches now offer unprecedented sensitivity to investigate liver macrophage biology. It also remains to be established if KC subpopulations exist at the transcriptomic single cell level. Interestingly, there are already few databases publically available such as *tabula muris* (45) or the mouse cell atlas (46) for mouse studies, but also human liver databases (31, 47). These databases offer the possibility for everyone, even without being equipped to perform single cell transcriptomics, to ask such questions on liver macrophages or others immune cell heterogeneity and screen for their own potential genes of interest. However, we cannot exclude the possibility that the strategies and technologies used in these studies did not allow for KC heterogeneity to be discovered: the number of cells sampled may have been too low, the sequencing depth may have been too superficial to reveal subtle and deeply hidden transcriptomic signatures, or the techniques allowing the isolation of KC could have introduced a bias of selection of a particular subset. A massive, single-cell study of KC heterogeneity at steady-state that includes a large enough cell population and a pipeline that permits deep sequencing and detects high numbers of genes is urgently needed.

Nevertheless, two recent studies discussed thereafter have exemplified how single cell transcriptomics can be used to gain insights into macrophage biology. The first one has clarified the crosstalk between endothelial cells, HSC and liver macrophages during the development of non-alcoholic steatohepatitis (NASH) (48). Authors used mainly a mouse model of NASH but also validated their observations in humans. Briefly, by doing single cell sequencing of liver non-parenchymal cells, they have observed that vascular signaling was dysregulated during NASH. They have also observed the emergence of a NASH-specific population of KC, expressing notably Trem2 and CD9. Very interestingly, it was also observed that these effects on endothelial cells and liver macrophages were orchestrated by HSCs via the expression of key secreted factors called “stellakines” (48). The second study, is focused on human liver cirrhosis (49). Authors have sequenced around 100,000 single cells, observed an heterogeneity in endothelial cells and the appearance of a Trem2+ CD9+ fibrotic macrophage population. They have also reconstructed the interactions between endothelial cells, macrophages and HSCs (49). These studies are interesting in many ways but focusing on macrophages, they are in line with another recent study describing a population of adipose tissue Trem2+ CD9+ macrophages that emerge during obesity and that regulate adipocyte hypertrophy and body fat accumulation (50). It argues for a pan-organ role of Trem2 signaling in tissue macrophages that is beyond the limits of hepatology but that is definitively interesting considering what is known on the central role of Trem2 in Alzheimer disease for example (51).

## Concluding Remarks

Herein, we discussed liver macrophage heterogeneity and how the most recent advances in single cell transcriptomics could be used to decipher liver macrophage biology. Clearly, last years of research have revealed an unexpected heterogeneity of liver macrophages, both at ontogeny and environmental levels. We now need to take into account this diversity in future studies focusing on liver diseases, and the use of the most recent and still evolving technologies such as the single cell transcriptomics will be crucial for this.

## Author Contributions

All authors listed have made a substantial, direct and intellectual contribution to the work, and approved it for publication.

### Conflict of Interest

The authors declare that the research was conducted in the absence of any commercial or financial relationships that could be construed as a potential conflict of interest.
